# Advances in cellular and molecular predatory biology of *Bdellovibrio bacteriovorus* six decades after discovery

**DOI:** 10.3389/fmicb.2023.1168709

**Published:** 2023-05-15

**Authors:** Ting F. Lai, Rhian M. Ford, Simona G. Huwiler

**Affiliations:** ^1^Department of Plant and Microbial Biology, University of Zurich, Zurich, Switzerland; ^2^School of Biosciences, University of Nottingham, Loughborough, United Kingdom

**Keywords:** predatory bacteria, *Bdellovibrio bacteriovorus*, molecular mechanisms, history, methods, antimicrobial resistance, predatory life cycle, cryo-electron tomography

## Abstract

Since its discovery six decades ago, the predatory bacterium *Bdellovibrio bacteriovorus* has sparked recent interest as a potential remedy to the antibiotic resistance crisis. Here we give a comprehensive historical overview from discovery to progressive developments in microscopy and molecular mechanisms. Research on *B. bacteriovorus* has moved from curiosity to a new model organism, revealing over time more details on its physiology and fascinating predatory life cycle with the help of a variety of methods. Based on recent findings in cryo-electron tomography, we recapitulate on the intricate molecular details known in the predatory life cycle including how this predator searches for its prey bacterium, to how it attaches, grows, and divides all from within the prey cell. Finally, the newly developed *B. bacteriovorus* progeny leave the prey cell remnants in the exit phase. While we end with some unanswered questions remaining in the field, new imaging technologies and quantitative, systematic advances will likely help to unravel them in the next decades.

## From curiosity to new model organism

**Figure 1:**
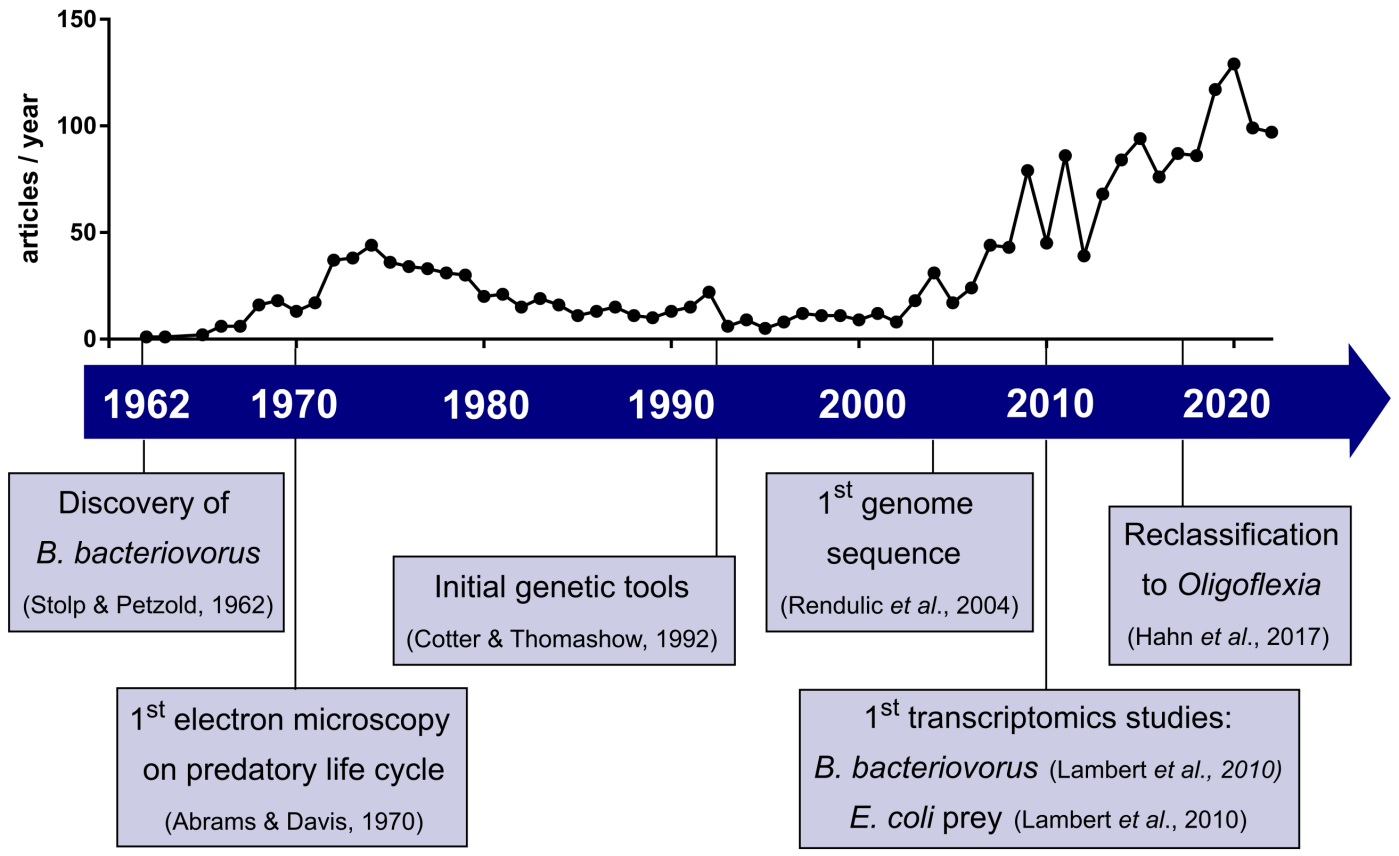
Development of *Bdellovibrio* research field in the last six decades with selected key events. Plot shows the number of articles published per year when searching *Bdellovibrio* on Web of Science^™^ until 2022.

What started as a serendipity-based discovery from a soil sample (Stolp and Petzold, 1962) was regarded for a long time as a curiosity in the microbial world. Recently, the research field of predatory bacteria and *Bdellovibrio* in particular, is gaining interest as a novel way to fight antimicrobial resistant pathogens and is growing rapidly due to methodological and technical advances over the last six decades. The number of published articles on *Bdellovibrio* per year, as indicated in Figure 1, has steadily increased in recent years—a trend coinciding with the rise of antimicrobial resistance studies.

**Figure 2:**
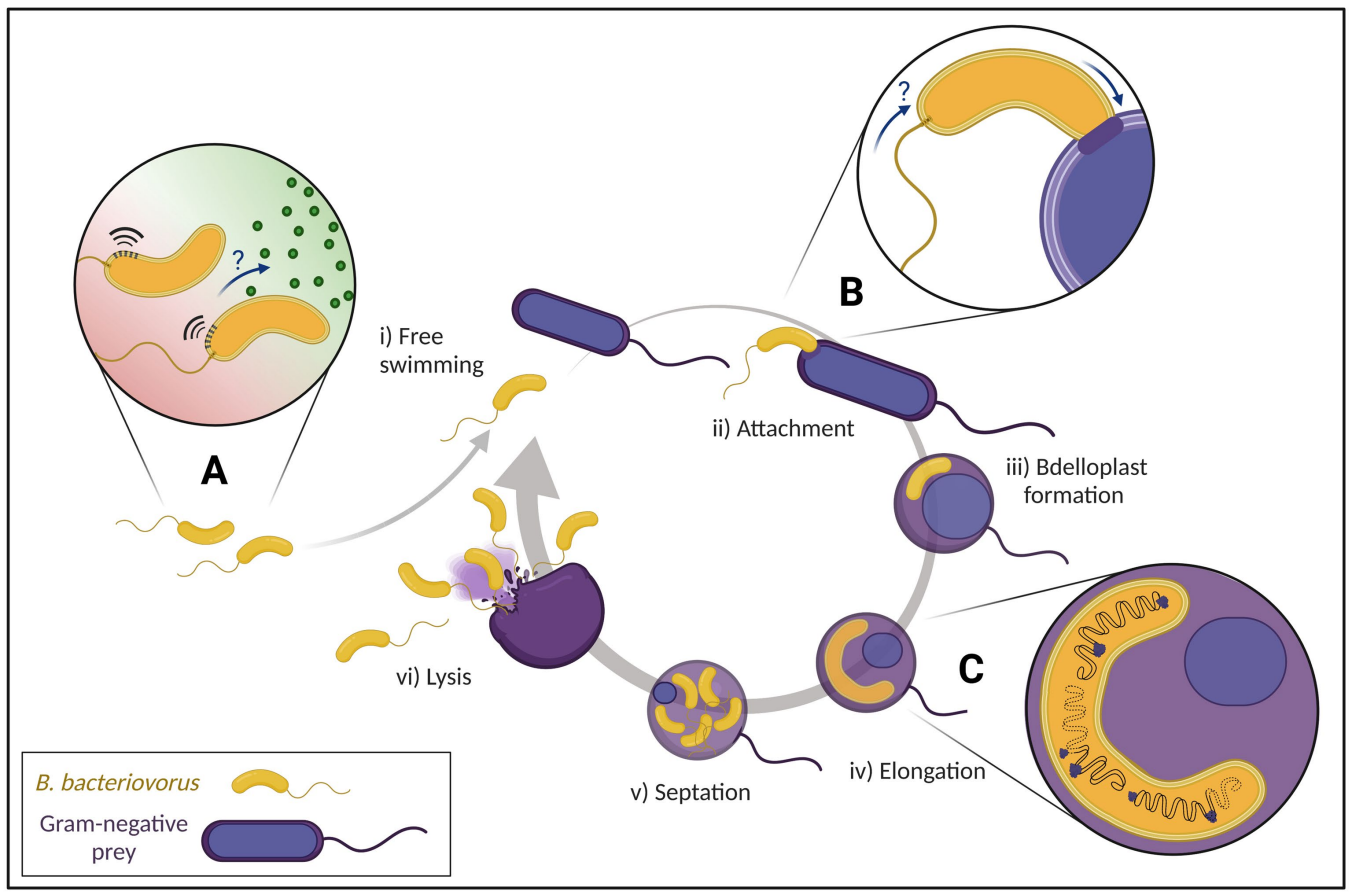
Predatory life cycle of *Bdellovibrio bacteriovorus* with focus on current advances of research. (i) Free swimming attack phase *B. bacteriovorus* move to regions with high prey density **(A)** by fast movement and possible chemotaxis (‘?’). (ii) After an initial reversible attachment to the prey, an irreversible attachment on suitable prey is formed **(B)** before entering the prey periplasm with suggested flagellar reabsorption (‘?’). (iii) After sealing the entry site behind itself, *B. bacteriovorus* establishes within the prey cell turning into an osmotically stable bdelloplast. (iv) The predator breaks down the prey cell contents to elongate while multiple asynchronous chromosomal replications take place **(C)**. (v) Once nutrient availability is depleted, *B. bacteriovorus* undergoes synchronous septation. (vi) Prey cell lysis releases progeny cells into the environment to repeat the predatory life cycle.

In 1962, whilst attempting to isolate bacteriophages, Stolp and Petzold discovered a small ‘parasitic’ organism which showed lytic activity against phytopathogenic *Pseudomonas* (Stolp and Petzold, 1962). But unlike bacteriophages, this new organism, named *Bdellovibrio bacteriovorus*, formed plaques after a longer incubation period and which increased in size (Stolp and Starr, 1963). Visualization using phase contrast microscopy showed that only Gram-negative species were susceptible to *B. bacteriovorus* (Starr and Baigent, 1966). Interestingly, a subset of *B. bacteriovorus* was shown to be able to grow independently of prey as host-independent (HI) strains (Shilo and Bruff, 1965). Further studies on how the predator invades bacterial prey cells led to an initial characterization of attachment and invasion (Varon and Shilo, 1968, 1969; Abram et al., 1974). Due to the lack of genetic tools and methods available, the mechanistic hypotheses proposed could not be investigated further. Early electron microscopy identified the morphology of *B. bacteriovorus* (Abram and Davis, 1970) as well as the predator–prey interactions in invasion and intracellular growth (Burnham et al., 1968). These studies set the foundations of the predatory life cycle we know today (Figure 2).

Based on this initial work, various aspects of the predator’s biology were explored deeper in the 1970s and 80s, prominently by three research groups: Samuel Conti’s group performed early chemotaxis experiments (LaMarre et al., 1977; Straley and Conti, 1977; Straley et al., 1979), and Sydney Rittenberg’s group investigated the biochemistry and metabolism of *B. bacteriovorus*—its breakdown and uptake of prey nutrients as well as the synthesis of its own macromolecules (Hespell et al., 1973, 1975; Kuenen and Rittenberg, 1975; Nelson and Rittenberg, 1981; Rosson and Rittenberg, 1981; Rayner et al., 1985). Furthermore, *B. bacteriovorus* population studies by Mazel Varon spearheaded research on the effect of predator–prey and predator-environment interactions on predatory ability (Varon and Zeigler, 1978; Varon, 1981; Varon and Shilo, 1981). After the initial boom of publications in the first two decades of *Bdellovibrio* research, the following years of the 1980s and 90s saw a decline in the number of publications in this field. It was not until the advent of the genomics era that re-sparked the interest in *Bdellovibrio* research. Cotter and Thomashow pioneered the first genetic work, successfully conjugating DNA from *E. coli* into *B. bacteriovorus* and proving *B. bacteriovorus* to be a genetically tractable model (Cotter and Thomashow, 1992). Undoubtedly, one of the most important milestones was in 2004, where Rendulic and colleagues published the first whole genome sequence of *B. bacteriovorus* HD100 (Rendulic et al., 2004). While most findings are based on *B. bacteriovorus* strains HD100 and 109J, numerous *B. bacteriovorus* genomes have been sequenced, isolated from a variety of different environments (Wurtzel et al., 2010; Hobley et al., 2012b; Oyedara et al., 2016). Recently, the order *Bdellovibrionales* was reclassified from the *Deltaproteobacteria* into the new class of *Oligoflexia* (Hahn et al., 2017). In the past 60 years, other genera and species of obligate predatory prokaryotes were discovered, generally referred to as BALOs (*Bdellovibrio* and like organisms). These BALOs have also been shown to be efficient against pathogenic bacteria and biofilms, but will not be discussed further in this mini review (Chanyi et al., 2016; Bratanis et al., 2020).

In 2010, the first transcriptomics study in *B. bacteriovorus* identified differentially expressed predatory and HI genes (Lambert et al., 2010a). Complementing this was the profiling of differentially regulated *E. coli* genes in response to the predator invasion (Lambert et al., 2010b). These genetic and transcriptomic approaches allowed a much broader view, thus supporting a deeper understanding of all stages of the predatory life cycle. Genetic manipulation coupled with early “omics” data and more recent structural determination studies of *B. bacteriovorus* proteins have revealed important functions and molecular mechanisms in different aspects of the predator’s biology (Lovering et al., 2011; Lerner et al., 2012; Lambert et al., 2015; Meek et al., 2019; Harding et al., 2020).

In the last decade, the therapeutic applications of *B. bacteriovorus* have become a prominent field of research including its use against antibiotic-resistant pathogens. The unique lifestyle of *B. bacteriovorus* has garnered interest as a predator against an impressive variety of pathogens (Dashiff and Kadouri, 2011; Iebba et al., 2014) and efficacy against Gram-negative biofilms (Kadouri and O’Toole, 2005; Núñez et al., 2005). Multiple animal models have been used to assess predatory ability alongside host safety *in vivo* (Atterbury et al., 2011; Romanowski et al., 2016; Shatzkes et al., 2016, 2017; Willis et al., 2016; Findlay et al., 2019). Concurrently, *in vitro* testing in sera and human cell lines has deepened the understanding of the human immune response towards *B. bacteriovorus* (Gupta et al., 2016; Monnappa et al., 2016; Baker et al., 2017; Raghunathan et al., 2019), a necessary step in developing *B. bacteriovorus* as a potential human therapeutic.

## Latest methods and technology on imaging and quantification

In the last couple of decades, many new methods and technologies, especially microscopy, have been essential for the analysis of *B. bacteriovorus.* Combining epifluorescence microscopy with genetic manipulations has revealed interesting morphological phenotypes alongside molecular mechanisms (Fenton et al., 2010a; Lambert et al., 2016; Banks et al., 2022). Further development of fluorescently labelled D-amino acids proved to be a useful tool in investigating peptidoglycan (PG) modifications by the predator using super-resolution microscopy (Kuru et al., 2017).

Electron microscopy has been used to resolve key structures like the flagellar motor in *B. bacteriovorus* (Morehouse et al., 2011; Chaban et al., 2018). Further advancements in electron microscopy techniques such as cryo-electron tomography (cryo-ET) supported the elucidation of novel structures within predator cells, to a resolution of individual proteins (Xu et al., 2019; Kaplan et al., 2022, Preprint). Alongside visualizing protein structures, the use of X-ray crystallography has generated models of individual *B. bacteriovorus* proteins, allowing elucidation of protein functions by evaluating interactions (Lambert et al., 2015; Cho et al., 2019; Meek et al., 2019).

Until recently, the enumeration of *Bdellovibrio* were based on double-layer overlay plates and plaque counting requiring three to four days (Rittenberg, 1982; Lambert and Sockett, 2008). Two new methods for *Bdellovibrio* spp. enumeration in under one hour have been reported in 2022. The resazurin assay relies on the detection of NADH produced by *B. bacteriovorus*, which transforms weakly fluorescent resazurin into the highly fluorescent resorufin (Jang et al., 2022). Alternatively, the SYBR Green assay quantifies total DNA content to determine predator concentration (Remy et al., 2022). The same authors developed an independent R-package, CuRveR, to analyze the decrease of prey OD_600nm_ opposite to the increase of fluorescence in the predator (when expressing a fluorescent cytoplasmic protein).

## Searching for prey

One major unknown in the *B. bacteriovorus* life cycle is how exactly the predator finds its prey (Figure 2A). Attack phase (AP) predator cells move up to 160 μm/s using a single polar flagellum (Lambert et al., 2006b), searching for suitable prey. *B. bacteriovorus* can pause swimming in prey limited environments and subsequently resume when prey bacteria are reintroduced (Sathyamoorthy et al., 2021), in a fine balance of energy conservation and prey detection for optimal predation.

Fast swimming speeds and altered motion make finding prey appear random, with limited evidence of any active sensing towards specific prey. A study of hydrodynamic forces from *B. bacteriovorus* movement showed that the self-generated disruption to the local liquid environment results in movement away from open spaces towards surfaces or obstacles. Prey hydrodynamic forces cause localization at surfaces, increasing encounter rate with *B. bacteriovorus* (Jashnsaz et al., 2017).

Combining speed with weak chemotaxis to amino acids (LaMarre et al., 1977) and high bacterial populations (regardless of prey suitability; Straley and Conti, 1977), *B. bacteriovorus* increases the chances of colliding with other bacteria. If the prey found is suitable, the initial reversible attachment becomes irreversible. Sequencing of the *B. bacteriovorus* HD100 genome (Rendulic et al., 2004) has revealed 20 methyl-accepting chemotaxis proteins (mcps) where multiple copies of genes encode the chemotaxis machinery alongside an absence of quorum sensing systems (Pasternak et al., 2013; Sester et al., 2020). Transposon mutants of mcps took longer to reduce prey cell numbers by half in liquid culture (Lambert et al., 2003), were unable to produce lytic plaques on prey cell lawns, and also had a significantly reduced ability to clear preformed biofilms (Medina et al., 2008). Cryo-ET data identifies chemosensory arrays at the flagellated pole of *B. bacteriovorus* in the attack and attachment phases (Borgnia et al., 2008; Butan et al., 2011; Kaplan et al., 2022, Preprint), which seem to be degraded and therefore absent in later stages (Kaplan et al., 2022, Preprint). While there clearly is a role for chemotaxis, it is not yet fully understood which exact part of the predatory cycle it impacts.

## Attaching to and entering the prey

During the attachment and invasion stages, several unique structures at the invasive pole of *B. bacteriovorus* are described. Abundantly found were fimbriae (Evans et al., 2007; Mahmoud and Koval, 2010), thought to be involved in the adhesion to facilitate invasion (Avidan et al., 2017). In nearly all AP cells, novel structures dubbed “rose-like complexes” were visualized with piliated and non-piliated Type IVa pili (T4aP) basal bodies often neighboring these structures (Kaplan et al., 2022, Preprint). It has been predicted that these T4aP are used to pull the prey cell closer (Evans et al., 2007; Mahmoud and Koval, 2010). Structural comparison (at macromolecular resolution) suggested similarities of the rose-like complexes to a tripartite efflux pump, possibly a Type I secretion system (Alav et al., 2021) and the non-piliated T4aP basal bodies to a Type II secretion system (Ghosal et al., 2019). Both structures may be implicated in effector secretion that modify the prey during invasion into an osmotically stable structure known as the bdelloplast (Rittenberg and Langley, 1975).

Kaplan et al., 2022 (Preprint) seems to detect by cryo-ET, that a majority of *B. bacteriovorus* HD100 cells seem to internalize the flagellum *via* a resorption mechanism once the predator is stably attached to the prey (Figure 2B). This differs from mechanisms found in other bacteria, which release their flagella under starvation, mechanical stress or by programmed ejection (Ferreira et al., 2019; Kaplan et al., 2019, 2021; Zhu et al., 2019; Zhuang et al., 2020). Why this occurs can only be speculated, perhaps as an energy conservation strategy employed by the predator (Smith and Chapman, 2010). However, this newest flagellar resorption report is opposed to a study with *B. bacteriovorus* 109 J, where the majority of predatory flagella were unshed (Lambert et al., 2006a).

## Development within the prey

Within the bdelloplast *B. bacteriovorus* transitions into a growth phase whereupon the prey contents are used for replication with prey-derived chemical cues driving the transcriptional changes required (Rotem et al., 2015). The genomic DNA of *B. bacteriovorus* has been shown to be tightly packed in a nucleoid, with ribosomes arranged at the edge (Borgnia et al., 2008; Butan et al., 2011; Kaplan et al., 2022, Preprint). Interestingly, the nucleoid area of 0.25 ± 0.03 μm^2^ is so dense during attack phase, it excludes freely diffusing monomeric proteins (Kaljević et al., 2021). Compaction, however, varies throughout the life cycle with relaxation of the nucleoid occurring between prey cell entry and DNA replication, resulting in multiple highly condensed nucleoids.

Subsequent cell division of *B. bacteriovorus* is remarkably different from typical bacterial cell division. Unlike many bacteria, *B. bacteriovorus* can follow non-binary division by elongating and dividing into a variable number of progenies correlating with prey cell size (Thomashow and Rittenberg, 1979; Fenton et al., 2010b; Kaljević et al., 2021). This filamentous cell division is controlled in part by DivIVA, responsible for progeny cell morphology with cell division coordinated by ParAB chromosomal partitioning systems (Milner et al., 2020). A recent study investigated *B. bacteriovorus* cell division by fluorescently labelling key components of the chromosome partitioning system ParABS (Kaljević et al., 2021). Imaging showed multiple asynchronous rounds of replication (Figure 2C) first initiated at the invasive pole, which is again different compared to the polarity of other monoflagellated bacteria like *Caulobacter crescentus* (Jensen and Shapiro, 1999) or *Vibrio cholerae* (Fogel and Waldor, 2004). The specific cues for staggered initiation of DNA replication and subsequent synchronous division are unknown.

## Exit phase

The mechanisms behind the exit phase by which the predator progeny leave the prey cell are not yet well understood, partially due to the technical difficulty of synchronizing *B. bacteriovorus* that exit the prey at a similar time. Initial hints on the exit mechanisms came from the study of secondary messenger signaling where cyclic GMP-AMP (cGAMP) has been shown to be critical in controlling gliding motility, the impairment of which leaves *B. bacteriovorus* HD100 stranded inside of the empty prey cell (Hobley et al., 2012a; Lowry et al., 2022). Furthermore, the deletion of two N-acetyl glucosamine (GlcNAc) deacetylase genes (*bd0468* and *bd3279*) left a “ghost” structure after predator exit, consisting mainly of PG and outer membrane porins (Lambert et al., 2016). Moreover, this predator mutant was delayed in escape time from prey cell remnants compared to wild type. While GlcNAc deacetylation of the prey PG happens during invasion/establishment by the predator (Lambert et al., 2016), this prey PG modification was shown to be important for DslA, a lysozyme that specifically acts on the GlcNAc deacetylated prey PG facilitating prey cell exit (Harding et al., 2020). *B. bacteriovorus ΔdslA* showed an increased time from division until leaving the prey remnants but did not abolish the exit process fully. Therefore DslA is likely part of a multifactorial system using different enzymes to break through the cell wall and outer membrane for exit from the prey cell. Fenton et al. observed that *B. bacteriovorus* progeny cells that escape through pores of the bdelloplast are consistently shorter than AP cells before infection. Further maturation/elongation occurs after release into the environment (Fenton et al., 2010a). While it was observed that gliding motility in *B. bacteriovorus* HD100 plays an integral part in exit from the prey cell, it is yet unknown how this mechanism interacts in the network of other established processes like enzyme secretion.

## Prospects for *Bdellovibrio bacteriovorus* research

Six decades after the discovery of *B. bacteriovorus,* researchers from 17 countries came together in virtual space for the first anniversary symposium ‘Celebrating sixty years of *Bdellovibrio* research’ to reflect on *Bdellovibrio* history and exchange the newest insights. Reflected by the modern way of meeting in an online format, the last six decades have transformed knowledge in many ways.

There are still many key questions on the cellular and molecular level to be solved with regards to the fascinating and useful predatory life cycle that *B. bacteriovorus* undergoes: (1) What structures and mechanisms gauge the suitability/specificity of the prey? (2) How can *B. bacteriovorus* possess such condensed genomic DNA, yet still be highly transcriptionally active and tightly regulated? (3) How does *B. bacteriovorus* gain access to the valuable prey cytoplasmatic contents? (4) What is the ultimate key signal that concludes *B. bacteriovorus* growth inside the prey and initiates division? (5) What are the multifactorial steps facilitating the exit of predator progeny cells from the prey cell remnants?

The expansion of novel technologies and improved methodologies has helped to gather data rapidly, expediting the sharing of new insights. Moving from light microscopy to fluorescence-assisted methods enabled more precise localization of structures, whilst the newest developments in cryo-ET enabled imaging at astonishing nm-resolution. While the latter resulted in new findings that we review here from Kaplan et al. (2022, Preprint), we expect that the coming decade will shed even more light into the precise mechanisms of how the predator interacts with its bacterial prey. Molecular structures determined by X-ray crystallography, fluorescence microscopy and bioassays have allowed a detailed view into the mechanistic side. Alphafold (Jumper et al., 2021; Varadi et al., 2022) has given researchers an additional powerful tool to help build hypotheses to elucidate the function of many uncharacterized predatory proteins. New developments in super-resolution microscopy and automated intelligence-assisted software help to analyze big microscopy data and gain a more in-depth understanding not only at the single cell level, but also on the level of different bacterial (prey/predator) populations. The newest trend in quantitative research will provide more knowledge on *B. bacteriovorus* behavior and (predator/prey) population dynamics with the help of mathematical modelling (de Dios Caballero et al., 2017; Hobley et al., 2020; Summers and Kreft, 2022), where models that dovetail nicely with wet lab experiments are of special value (Hobley et al., 2020). With these new tools in hand, the research community will certainly gain a better understanding of the complex interactions within microbial communities. The technological advances on different ‘omics’ technologies in the last two decades help us to understand the complex interactions of bacterial predator and prey. With a multitude of transcriptomics studies (Lambert et al., 2010a; Karunker et al., 2013; Dwidar et al., 2017), there is certainly much to be gained from applying further holistic technologies.

While research on *B. bacteriovorus* has sometimes learned from advances made in the related predator *Myxococcus xanthus* (Seef et al., 2021), it has since developed into a growing research field on its own. The newest technologies equip us with valuable tools to resolve the many remaining questions in the future, which is an important prerequisite to leverage *B. bacteriovorus* in the fight against antimicrobial resistance.

## Author contributions

TL, RF, and SH wrote the manuscript. SH made Figure 1 (with Inkscape). RF made Figure 2 (Created with BioRender.com). All authors contributed to the article and approved the submitted version.

## Funding

This work was funded by the Swiss National Science Foundation Ambizione Grant PZ00P3_193401 to TL and SH. RF was supported by the Wellcome Trust Antimicrobials and Antimicrobial Resistance DTP studentship 108876/B/15/Z.

## Conflict of interest

The authors declare that the research was conducted in the absence of any commercial or financial relationships that could be construed as a potential conflict of interest.

## Publisher’s note

All claims expressed in this article are solely those of the authors and do not necessarily represent those of their affiliated organizations, or those of the publisher, the editors and the reviewers. Any product that may be evaluated in this article, or claim that may be made by its manufacturer, is not guaranteed or endorsed by the publisher.
